# Age and Late Recurrence in Young Patients With ER–Positive, *ERBB2*-Negative Breast Cancer

**DOI:** 10.1001/jamanetworkopen.2024.42663

**Published:** 2024-11-07

**Authors:** Dong Seung Shin, Janghee Lee, Eunhye Kang, Dasom Noh, Jong-Ho Cheun, Jun-Hee Lee, Yeongyeong Son, Soong June Bae, Seok Won Kim, Jeong Eon Lee, Jonghan Yu, Byung-Joo Chae, Sunyoung Kwon, Han-Byoel Lee, Sung Gwe Ahn, Jai Min Ryu

**Affiliations:** 1 Division of Breast Surgery, Department of Surgery, Samsung Medical Center, Sungkyunkwan University of Medicine, Seoul, Republic of Korea; 2 Department of Surgery, Dongtan Sacred Heart Hospital, Hallym University, Dongtan, Republic of Korea; 3 Department of Medicine, Yonsei University Graduate School, Seoul, Republic of Korea; 4 Department of Surgery, Seoul National University College of Medicine, Seoul, Republic of Korea; 5 Cancer Research Institute, Seoul National University, Seoul, Republic of Korea; 6 Department of Information Convergence Engineering, College of Information and Biomedical Engineering, Pusan National University, Busan, Republic of Korea; 7 Department of Surgery, Seoul Metropolitan Government–Seoul National University Boramae Medical Center, Seoul, Republic of Korea; 8 Department of Surgery, Soonchunhyang University College of Medicine, Soonchunhyang University Hospital, Seoul, Republic of Korea; 9 Department of Surgery, Gangnam Severance Hospital, Yonsei University College of Medicine, Seoul, Republic of Korea; 10 School of Biomedical Convergence Engineering, College of Information and Biomedical Engineering, Pusan National University, Yangsan, Republic of Korea; 11 Center for Artificial Intelligence Research, Pusan National University, Busan, Republic of Korea; 12 Biomedical Research Institute, Seoul National University Hospital, Seoul, Republic of Korea; 13 Institute for Breast Cancer Precision Medicine, Gangnam Severance Hospital, Yonsei University College of Medicine, Seoul, Republic of Korea

## Abstract

**Importance:**

Young patients with breast cancer with estrogen receptor (ER)–positive, *ERBB2*-negative tumors have a poor prognosis. Understanding factors influencing late recurrence is crucial for improving management and outcomes.

**Objective:**

To determine whether age is an independent factor associated with late distant recurrence (DR) in young patients with ER-positive, *ERBB2*-negative cancers without distant metastasis within 5 years from surgery.

**Design, Setting, and Participants:**

This multicenter retrospective cohort study analyzed clinical records of patients with breast cancer who underwent surgery from January 2000 to December 2011 with at least 5 years of follow-up. The study was conducted at Samsung Medical Center, Gangnam Severance Hospital, and Seoul National University Hospital, including patients aged 45 years or younger with ER-positive, *ERBB2*-negative tumors, no DR within 5 years after surgery, no neoadjuvant chemotherapy, and at least 2 years of endocrine therapy. The data analysis period was from January 4, 2023, to March 21, 2024.

**Exposure:**

Age, grouped as 21 to 35 years, 36 to 40 years, and 41 to 45 years.

**Main Outcomes and Measures:**

The primary outcome was the incidence of late DR at 5 to 10 years after surgery. Survival outcomes, including late distant metastasis–free survival (DMFS), were evaluated in different age groups.

**Results:**

Among 2772 patients included, 370 (13.3%) were aged 21 to 35 years, 885 (31.9%) were aged 36 to 40 years, and 1517 (54.7%) were aged 41 to 45 years. The median (range) follow-up was 10.8 (5.0-21.4) years. The youngest group had a poorer histologic grade (eg, histologic grade 3: 107 patients aged 21-35 years [28.9%]; 149 patients aged 36-40 years [16.8%]; 273 patients aged 41-45 years [18.0%]) and more frequent chemotherapy (307 patients aged 21-35 years [83.0%]; 697 patients aged 36-40 years [78.8%]; 1111 patients aged 41-45 years [73.2%]). The youngest patients had significantly worse rates of locoregional recurrence–free survival (patients aged 21-35 years, 90.1% [95% CI, 86.8%-93.3%]; patients aged 36-40 years, 94.6% [95% CI, 93.0%-96.2%]; patients aged 41-45 years, 97.7% [95% CI, 96.9%-98.5%]), disease-free survival (patients aged 21-35 years, 79.3% [95% CI, 75.0%-83.9%]; patients aged 36-40 years, 88.7% [95% CI, 86.5%-91.0%]; patients aged 41-45 years, 94.4% [95% CI, 93.2%-95.7%]), and late DMFS (patients aged 21-35 years, 89.3% [95% CI, 86.0%-92.9%]; patients aged 36-40 years: 94.2% [95% CI, 92.5%-95.9%]; patients aged 41-45 years: 97.2% [95% CI, 96.3%-98.1%]) but not overall survival (patients aged 21-35 years, 96.9% [95% CI, 95.0%-98.9%]; patients aged 36-40 years, 98.2% [95% CI, 97.2%-99.2%]; patients aged 41-45 years, 98.9% [95% CI, 98.3%-99.5%]). Multivariable analysis showed lower hazard for late DR in the older groups compared with the youngest group (age 36-40 years: hazard ratio, 0.53; 95% CI, 0.34-0.82; *P* = .001; age 41-45 years: hazard ratio, 0.30; 95% CI, 0.20-0.47; *P* < .001).

**Conclusions and Relevance:**

In this retrospective cohort study, age was an independent factor associated with late DR in young patients with ER-positive, *ERBB2*-negative breast cancer. Younger age was associated with worse locoregional recurrence–free survival, disease-free survival, and late DMFS, highlighting the importance of long-term monitoring and potential for personalized treatment approaches based on age, particularly for younger patients with ER-positive, *ERBB2*-negative breast cancer.

## Key Points

**Question:**

Is age an independent factor associated with late distant recurrence among young patients with breast cancer with estrogen receptor (ER)–positive, *ERBB2*-negative tumors?

**Findings:**

In this cohort study of 2772 patients aged 45 years or younger with ER-positive, *ERBB2*-negative breast cancer and no distant metastasis within 5 years after surgery, younger age was significantly associated with worse disease-free survival, locoregional recurrence-free survival, and late distant metastasis–free survival.

**Meaning:**

These findings suggest that age was an independent risk factor associated with late distant recurrence in young patients with ER-positive, *ERBB2*-negative breast cancer, indicating the need for tailored management and follow-up strategies, particularly for younger patients.

## Teaser

This cohort study examines the association of age with late distant recurrence in patients aged 45 years or younger with estrogen receptor (ER)–positive, *ERBB2*-negative breast cancer.

## Introduction

Patients with breast cancer who are young at diagnosis generally have poorer survival than older groups.^1,2^ Several prospective and retrospective studies have reported that breast cancers diagnosed at a younger age have more aggressive pathologic features. They are typically diagnosed at a larger size, have greater lymph node involvement, are poorly differentiated, and are often hormone receptor–negative disease. Molecular profiling also shows that younger patients with breast cancer have more basal-like tumors. Additionally, young patients with breast cancer are known to have more frequent hereditary breast cancer syndromes, which are associated with a particularly poor prognosis in luminal-like disease.^3,4^

Hormone receptor–positive breast cancer is the most common subtype of breast cancer, accounting for approximately 60% to 75% of all breast cancers, and is associated with a relatively good prognosis compared with other subtypes.^5^ Hormone receptor–positive, *ERBB2*-negative breast cancer is associated with a higher risk of late recurrence compared with other subtypes.^6^ A meta-analysis of 62 923 patients with estrogen receptor (ER)–positive breast cancer enrolled in 88 studies who were followed up for death and recurrence after 5 years of adjuvant endocrine therapy (ET) showed a cumulative risk of recurrence ranging from as low as 10% to as high as approximately 40%, depending on stage, from 5 to 20 years after diagnosis.^7,8,9,10^

Although several studies have shown no significant differences in survival according to age in hormone receptor–negative breast cancer, it has been reported that hormone receptor–positive breast cancer diagnosed at a younger age has a poorer survival outcome.^11,12^ Because young patients with hormone receptor–positive, *ERBB2*-negative breast cancer have a relatively long life expectancy, the cumulative risk of late recurrence after 5 years in younger patients is greater than that of older patients, which should be considered in the treatment of young patients with breast cancer. Despite many studies analyzing differences in survival based on patient age, there is a shortage of research on late distant recurrence (DR) after 5 years, specifically in young patients with breast cancer who have remained recurrence-free for the initial 5 years after surgery.

This study aimed to analyze whether younger patients had a worse prognosis for late DR in a group of patients with ER-positive, *ERBB2*-negative breast cancer who were recurrence-free within 5 years after surgery. We conducted a factor analysis to investigate whether age at diagnosis of breast cancer was an independent factor associated with late DR in young patients with breast cancer.

## Methods

### Study Population and Data Collection

This cohort study was approved by the institutional review boards of each participating institution, which followed the ethical principles of the Declaration of Helsinki. The requirement for informed consent was waived because the study was performed retrospectively. This study is reported according to the Strengthening the Reporting of Observational Studies in Epidemiology (STROBE) reporting guideline.

**Figure 1.**
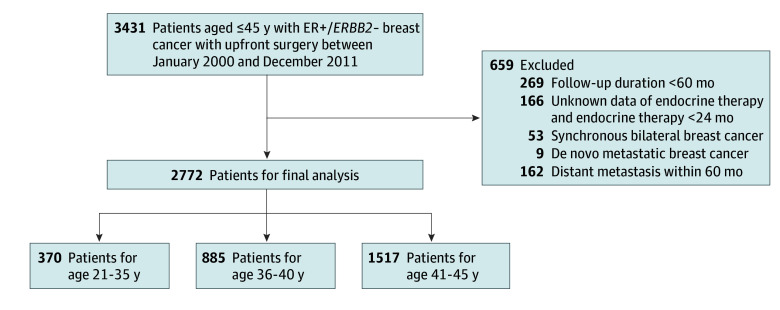
Schematic Diagram of the Study The patients with local recurrence within 60 months of follow-up were included. ER+ indicates estrogen receptor–positive, *ERBB2*−, *ERBB2*-negative.

We retrospectively reviewed data from patients who underwent surgery for breast cancer between January 2000 and December 2011 at 3 major institutions in Korea: Samsung Medical Center, Gangnam Severance Hospital, and Seoul National University Hospital. We only included patients with ER-positive, *ERBB2*-negative breast cancer who had no DR within 5 years from the operation date and who were aged 45 years or younger. Women with bilateral cancers, neoadjuvant chemotherapy, less than 24 months of ET, and ER-negative or unknown disease type were excluded. The patients with locoregional recurrence (LRR) within 5 years were included. (Figure 1).

Patients were stratified into 3 groups based on age at breast cancer diagnosis. The clinicopathological characteristics and oncologic outcomes of these 3 groups were compared. The primary end point of this study was late DR in young patients with ER-positive, *ERBB2*-negative breast cancer. We focused on patients aged 45 years or younger, although the definition of *young* in breast cancer studies varies, with cutoffs at 30, 35, 40, and 45 years. Han et al^1^ suggest 35 years is a reasonable cutoff age to define *young* for patients with breast cancer based on data from the Korean Breast Cancer Society Registry.^1^ Additionally, recent studies using the Surveillance, Epidemiology, and End Results (SEER) database found that patients younger than 40 years had significantly worse survival rates compared with those aged 40 to 60 years.^13,14^ Based on these findings, we divided our cohort into 3 age groups at diagnosis: 21 to 35 years, 36 to 40 years, and 41 to 45 years.

### Hormone Receptors and Adjuvant Treatment

Each institution reviewed pathologic data in their medical records for ER, progesterone receptor, and *ERBB2* status, as evaluated by their own analysis methods. Based on this information, we only included patients with ER-positive and *ERBB2*-negative breast cancers, regardless of progesterone receptor status.

After surgery, all patients in this study received adjuvant ET. If the patient was receiving adjuvant chemotherapy, ET was started after chemotherapy. Based on clinical and pathologic information, some patients received gonadotropin-releasing hormone agonist treatment as ovarian function suppression (OFS), and some patients had extended ET for more than 5 years.

### Definitions of Recurrence and Survival

For this study, LRR of breast cancer included local recurrence in the ipsilateral chest wall, skin, subcutaneous tissue, or pectoralis muscle, while regional recurrence was defined as occurring within the ipsilateral axillary, supraclavicular, internal mammary, or infraclavicular lymph nodes. DR was used to describe any recurrence in areas not included in LRR.

LRR-free survival (LRFS) was defined as the duration from surgery until the occurrence of the first LRR. Distant metastasis–free survival (DMFS) was defined as the duration from surgery until the occurrence of the first DR. Late DMFS in this study was defined as the period from 5 years after primary surgery until the first DR occurred. Disease-free survival (DFS) refers to the period from surgery until the first recurrence of the disease. Overall survival (OS) was defined as the time from surgery until death from any cause. For survival outcomes of the patients without recurrence or death during the follow-up period, LRFS, DFS, late DMFS, and OS were calculated as the time between surgery and the last visit date to the hospital. Information on recurrence events was acquired from the review of electronic medical records, and survival data were collected from the electronic medical records and the Korean National Statistical Office database.

### Statistical Analysis

We used Kruskal-Wallis test for continuous variables and χ^2^ or Fisher exact test for categorical variables to compare patient characteristics. Kaplan-Meier curves were generated for LRFS, DFS, DMFS, and OS with the corresponding outcomes of log-rank tests. Cox proportional hazards regression models were used for univariable and multivariable analyses for identifying factors associated with late DM in the cohort. The proportional hazard assumption was checked using Schoenfeld residuals. Multicollinearity in multivariable analysis was checked using variance inflation factor with threshold at 5.0.^15^ All statistical analyses were executed using SAS software version 9.4 (SAS Institute) and R software version 4.2.2 (R Project for Statistical Computing). *P* values were 2-sided, and *P* < .05 defined statistical significance. The data analysis period was from January 4, 2023, to March 21, 2024.

## Results

### Patient Characteristics

Of 2772 patients analyzed in this study, 370 (13.3%) were aged 35 years or younger, 885 (31.9%) were aged 36 to 40 years, and 1517 (54.7%) were aged 41 years or older. The median (range) age of all patients in this study was 41 (21-45) years, and the median (range) follow-up duration was 10.8 (5.0-21.4) years. All patients received adjuvant ET for at least 2 years, mostly with tamoxifen (2702 patients [97.5%]), with only 60 patients (2.2%) receiving toremifene and 10 patients (0.3%) receiving aromatase inhibitors with OFS. There were 2496 patients (90.0%) received ET for 5 years or more, and 276 patients (10.0%) in this study cohort received from 2 to 5 years of ET. There were 743 patients (26.8%) who extended ET with tamoxifen or aromatase inhibitors for more than 5 years. The median (IQR) duration of ET of all patients was 60 (59-71) months. Regarding adjuvant chemotherapy and radiotherapy, 2115 patients (76.3%) underwent chemotherapy, while 2003 patients (72.3%) underwent radiotherapy after surgery.

The clinicopathologic and treatment characteristics of each group are shown in Table 1. The youngest patients in this study (age 21-35 years) had significantly larger proportions of high nuclear grade and histologic grade tumors than the older groups (eg, histologic grade 3: 107 patients aged 21-35 years [28.9%]; 149 patients aged 36-40 years [16.8%]; 273 patients aged 41-45 years [18.0%]). Compared with older patients, the youngest patients were more likely to have received adjuvant chemotherapy (307 patients aged 21-35 years [83.0%]; 697 patients aged 36-40 years [78.8%]; 1111 patients aged 41-45 years [73.2%]). There was a difference in duration of ET among groups, with a median (IQR) of 60.0 (58.0-62.0) months for patients aged 21 to 35 years, 60.0 (59.0-68.0) months for patients aged 36 to 40 years, and 60.0 (59.0-81.0) months for patients aged 41 to 45 years. We observed that 59 patients aged 21 to 35 years (15.9%), 70 patients aged 36 to 40 years (7.9%), and 112 patients aged 41 to 45 years (7.4%) received less than 5 years of ET. There were no significant differences in T stage, N stage, progesterone receptor status, Ki-67 status, operation types, or radiotherapy among the 3 groups (Table 1).

**Table 1.** Baseline Characteristics of the 3 Groups

Characteristics	Patients by age at diagnosis, No. (%)			Overall *P* value
	21-35 y (n = 370)	36-40 y (n = 885)	41-45 y (n = 1517)	
Pathologic T stage				
T1	214 (57.8)	513 (58.0)	896 (59.1)	.97
T2	138 (37.3)	332 (37.5)	556 (36.7)	
T3-T4	18 (4.9)	40 (4.5)	65 (4.3)	
Pathologic N stage				
N0	222 (60.0)	517 (58.4)	974 (64.2)	.09
N1	107 (28.9)	254 (28.4)	387 (25.5)	
N2	27 (7.3)	86 (9.7)	110 (7.3)	
N3	14 (3.8)	28 (3.2)	46 (3.0)	
Nuclear grade				
1	52 (14.1)	119 (13.4)	257 (16.9)	.001
2	203 (54.9)	553 (62.5)	907 (59.8)	
3	113 (30.5)	194 (21.9)	327 (21.6)	
Unknown	2 (0.5)	19 (2.1)	26 (1.7)	
Histologic grade				
1	66 (17.8)	211 (23.8)	411 (27.1)	<.001
2	185 (50.0)	476 (53.8)	763 (50.3)	
3	107 (28.9)	149 (16.8)	273 (18.0)	
Unknown	12 (3.2)	49 (5.5)	70 (4.6)	
Progesterone receptor				
Positive	331 (89.5)	799 (90.3)	1399 (92.2)	.13
Negative	39 (10.5)	85 (9.6)	118 (7.8)	
Unknown	0 (0.0)	1 (0.1)	0 (0.0)	
Ki-67				
<20%	222 (60.0)	589 (66.6)	1022 (67.4)	.08
≥20%	73 (19.7)	158 (17.9)	240 (15.8)	
Unknown	75 (20.3)	138 (15.6)	255 (16.8)	
Breast operation				
BCS	230 (62.2)	551 (62.3)	977 (64.4)	.50
TM	140 (37.8)	334 (37.7)	540 (35.6)	
Axillary operation				
SLNB	188 (50.8)	447 (50.5)	837 (55.2)	.06
ALND	182 (49.2)	438 (49.5)	680 (44.8)	
Adjuvant chemotherapy				
Yes	307 (83.0)	697 (78.8)	1111 (73.2)	<.001
No	63 (17.0)	188 (21.2)	405 (26.7)	
Unknown	0 (0.0)	0 (0.0)	1 (0.1)	
Radiotherapy				
Yes	257 (69.5)	643 (72.7)	1103 (72.7)	.50
No	111 (30.0)	242 (27.3)	411 (27.1)	
Unknown	2 (0.5)	0 (0.0)	3 (0.2)	
Extended ET				
Yes	78 (21.1)	224 (25.3)	441 (29.1)	.004
No	292 (78.9)	661 (74.7)	1076 (70.9)	
Duration of ET, median (IQR), mo	60.0 (58.0-62.0)	60.0 (59.0-68.0)	60.0 (59.0-81.0)	<.001
Type of ET				
Tamoxifen	367 (99.2)	867 (98.0)	1468 (96.8)	.09
Toremifene	2 (0.5)	16 (1.8)	42 (2.8)	
Aromatase inhibitors with OFS	1 (0.3)	2 (0.2)	7 (0.4)	

Abbreviations: ALND, axillary lymph node dissection; BCS, breast conserving surgery; ET, endocrine therapy; OFS, ovarian function suppression; SLNB, sentinel lymph node biopsy; TM, total mastectomy.

### Survival Outcomes According to Age

**Figure 2.**
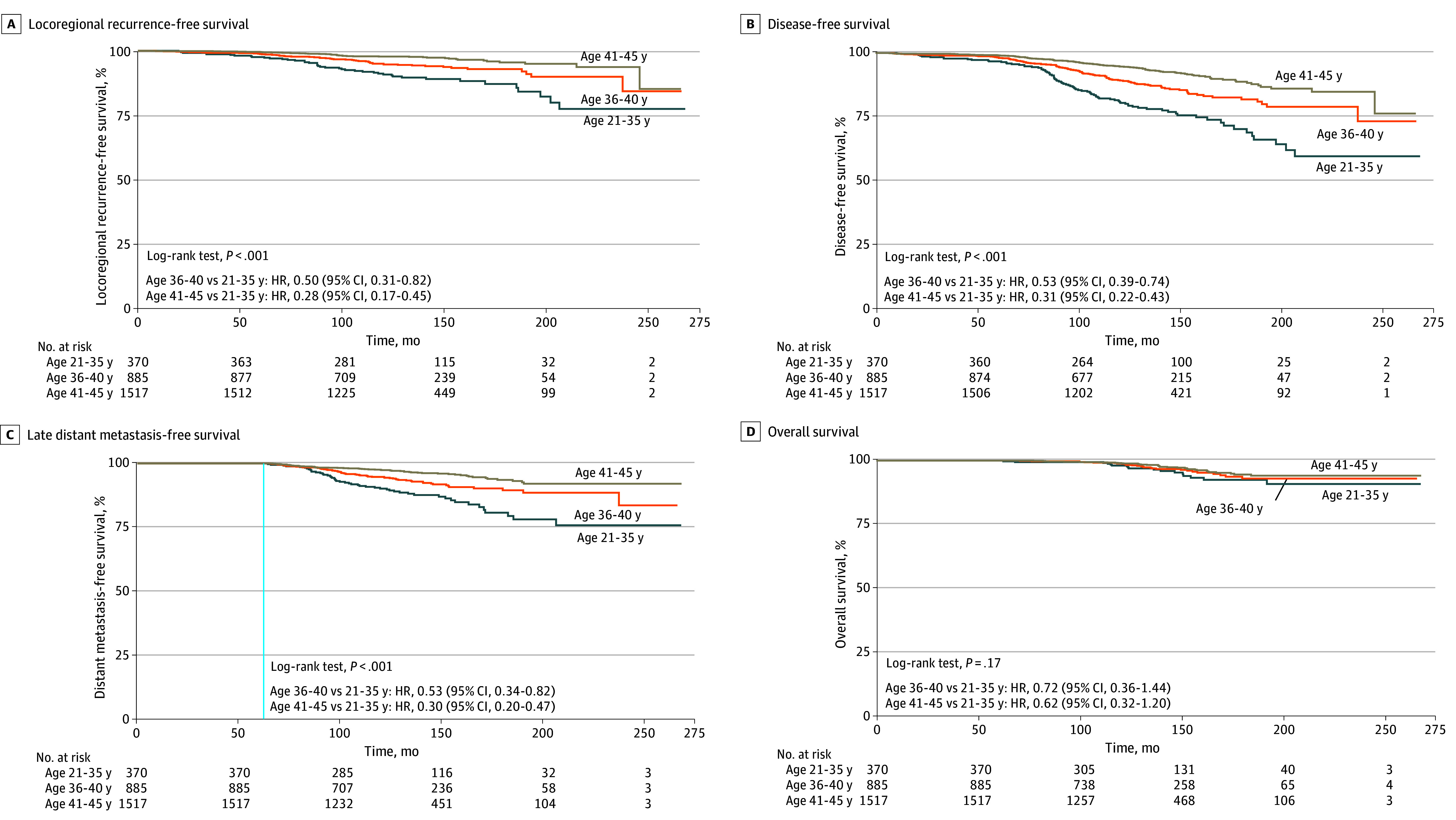
Kaplan-Meier Survival Curves for Recurrence-Free Survival and Overall Survival The patients with locoregional recurrence within 5 years were included in this study. Late distant metastasis-free survival in this study was defined as the period from 5 years after primary surgery until the first distant metastasis occurred. HR indicates hazard ratio.

The 10-year rates for LRFS, DFS, and OS were increased across age groups, with the youngest group exhibiting the lowest rates for LRFS (patients aged 21-35 years, 90.1% [95% CI, 86.8%-93.3%]; patients aged 36-40 years, 94.6% [95% CI, 93.0%-96.2%]; patients aged 41-45 years, 97.7% [95% CI, 96.9%-98.5%]), DFS (patients aged 21-35 years, 79.3% [95% CI, 75.0%-83.9%]; patients aged 36-40 years, 88.7% [95% CI, 86.5%-91.0%]; patients aged 41-45 years, 94.4% [95% CI, 93.2%-95.7%]), and OS (patients aged 21-35 years, 96.9% [95% CI, 95.0%-98.9%]; patients aged 36-40 years, 98.2% [95% CI, 97.2%-99.2%]; patients aged 41-45 years, 98.9% [95% CI, 98.3%-99.5%]). The 10-year late DMFS rates, calculated from 5 years after primary surgery, showed an increase across age groups. Notably, the youngest patients exhibited the lowest rate of DMFS, at 89.3% (95% CI, 86.0%-92.9%), followed by patients aged 36 to 40 years, at 94.2% (95% CI, 92.5%-95.9%), and patients aged 41 to 45 years, at 97.2% (95% CI, 96.3%-98.1%). Kaplan-Meier survival curves for the different age groups for LRFS, DFS, late DMFS, and OS are shown in Figure 2. In this study, 31 patients who experienced LRR within the first 5 years received appropriate resection and adjuvant treatment with curative intent were included. Kaplan-Meier survival curves for late DMFS according to early LRR are shown in eFigure 2 in Supplement 1; however, given the small number of patients experiencing LRR relative to those who did not, the comparison resulted in significant data imbalance, rendering the statistical analysis less meaningful. Additionally, when analyzing late DMFS excluding the 31 patients with early LRR, the results were not significantly different from those obtained when including patients with early LRR (eFigure 3 in Supplement 1).

### Cox Proportional Hazards Regression Analysis for Survival Outcomes

On univariable analysis, younger age, higher pathologic T and N stages, higher histologic grade, total mastectomy (compared with breast-conserving surgery), and axillary lymph node dissection (compared with sentinel lymph node biopsy) were significantly associated with late DR (Table 2). In multivariable analyses with adjustment for variables, including age group, pathologic T and N stages, histologic grade, types of breast surgery and axillary surgery, younger age was a significant independent factor associated with LRFS, DFS, and late DMFS. However, young age was not a factor associated with OS in this study. The adjusted hazard for late DR was lower in older groups than in the youngest group (age 21-35 vs 36-40 years: adjusted hazard ratio [aHR], 0.53; 95% CI, 0.34-0.82; *P* = .001; age 21-35 vs 41-45 years: aHR, 0.30; 95% CI, 0.20-0.47; *P* < .001) (Table 2). In multivariable analysis with the same covariates and treating age as a continuous variable, a 1-year increase in age at breast cancer diagnosis was associated with a 9% lower risk of late DR (aHR, 0.91; 95% CI, 0.88-0.93; *P* < .001) (eTable 1 in Supplement 1).

**Table 2.** Univariable and Multivariable Analyses for Late Distant Recurrence

Variable	Univariable		Multivariable	
	HR (95% CI)	*P* value	HR (95% CI)	*P* value
Age, y^a^				
21-35	1 [Reference]	NA	1 [Reference]	NA
36-40	0.54 (0.35-0.83)	.001	0.53 (0.34-0.82)	.001
41-45	0.28 (0.18-0.44)	<.001	0.30 (0.20-0.47)	<.001
Pathologic T stage^b^				
T1	1 [Reference]	NA	1 [Reference]	NA
T2	2.62 (1.79-3.82)	<.001	1.66 (1.10-2.49)	.006
T3-T4	2.95 (1.48-5.88)	<.001	1.39 (0.66-2.94)	.33
Pathologic N stage^c^				
N0	1 [Reference]	NA	1 [Reference]	NA
N1	2.76 (1.78-4.30)	<.001	1.55 (0.85-2.82)	.08
N2	4.59 (2.72-7.73)	<.001	2.09 (1.05-4.15)	.01
N3	3.87 (1.75-8.55)	<.001	1.65 (0.65-4.14)	.20
Nuclear grade^d^				
1	1 [Reference]	NA	NA	NA
2	1.82 (1.06-3.13)	.03	NA	NA
3	1.80 (0.99-3.24)	.05	NA	NA
Histologic grade^e^				
1	1 [Reference]	NA	1 [Reference]	NA
2	2.64 (1.47-4.76)	<.001	1.89 (1.04-3.42)	.02
3	3.30 (1.75-6.22)	<.001	1.86 (0.97-3.58)	.03
Progesterone receptor				
Positive	1 [Reference]	.17	NA	NA
Negative	1.37 (0.87-2.15)		NA	NA
Breast operation				
BCS	1 [Reference]	<.001	1 [Reference]	.02
TM	2.21 (1.62-3.01)		1.48 (1.06-2.06)	
Axillary operation				
SLNB	1 [Reference]	<.001	1 [Reference]	.07
ALND	3.47 (2.40-5.00)		1.65 (0.97-2.80)	

Abbreviations: ALND, axillary lymph node dissection; BCS, breast conserving surgery; HR, hazard ratio; NA, not applicable; SLNB, sentinel lymph node biopsy; TM, total mastectomy.

^a^ Overall unadjusted *P* < .001; adjusted *P* < .001.

^b^ Overall unadjusted *P* < .001; adjusted *P* = .02.

^c^ Overall unadjusted *P* < .001; adjusted *P* = .09.

^d^ Overall unadjusted *P* = .09.

^e^ Overall unadjusted *P* < .001; adjusted *P* = .05.

In contrast to our expectations, pathologic N stage was not an independent factor associated with late DR on multivariable analysis, although there was an association between pathologic N stage and late DR in the univariable analysis. However, survival analyses according to pathologic N stages found that increased N stage was associated with decreased DFS, late DMFS, and OS, whereas LRFS did not show statistically significant differences according to pathologic N stage (eFigure 1 in Supplement 1).

**Figure 3.**
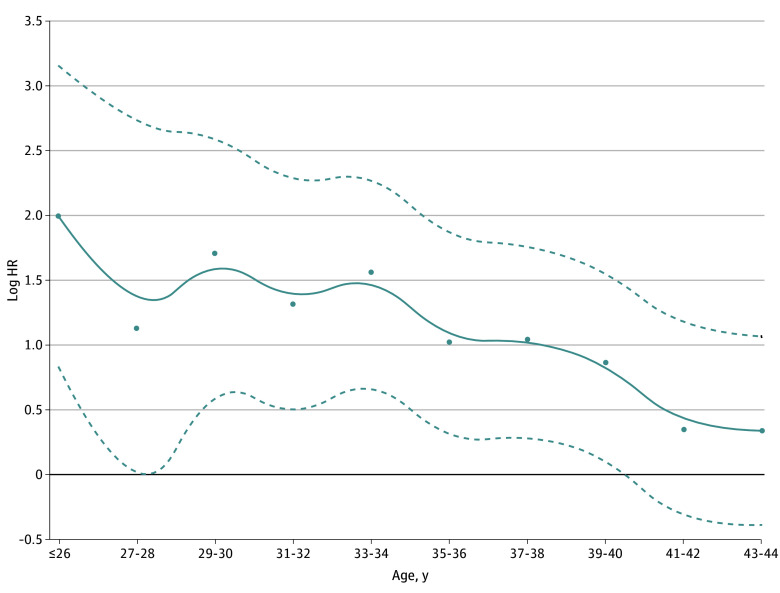
Breast Cancer Onset Age and Hazard of Late Distant Recurrence Analysis used Cox proportional hazards regression, with age 45 years as the reference group. The solid line indicates the smoothed hazard ratio (HR) line using spline functions; dashed lines, 95% CI; dots, individual HR estimates.

To investigate the association between late DR and age, we stratified patients into 11 age groups at 2-year intervals, specifically: 21 to 26, 27 to 28, 29 to 30, 31 to 32, 33 to 34, 35 to 36, 37 to 38, 39 to 40, 41 to 42, 43 to 44, and 45 years (eTable 2 in Supplement 1). We then conducted Cox proportional hazards regression analysis using these age groups, with the patients aged 45 years at diagnosis serving as the reference category (eTable 3 in Supplement 1). The smoothed HR line, derived using spline functions, indicated a trend toward a decreased risk of late DR with increasing age (Figure 3).

## Discussion

This large, multicenter, retrospective cohort study included 2772 patients with ER-positive, *ERBB2*-negative breast cancer and had a long median follow-up duration of 10.8 years. Our study results suggest that younger age was an independent factor associated with late DR in patients with ER-positive, *ERBB2*-negative breast cancer. Among the patients aged 45 years or younger, patients aged 35 years or younger had worse oncologic outcomes compared with older groups for LRFS, DFS, and late DMFS but not for OS.

Previous research has consistently shown a poorer prognosis for younger patients with ER-positive, *ERBB2*-negative breast cancer.^16^ Several population-based studies have highlighted young age as an independent risk factor associated with unfavorable outcomes specific to breast cancer. However, those studies concluded that younger age at diagnosis had more frequent association with clinicopathologic features and gene expression for worse prognosis, including lower ER positivity, larger tumors, higher *ERBB2* overexpression, lymph node positivity, and higher-grade tumors.^17,18,19^ Unlike previous studies, this study focused on ER-positive, *ERBB2*-negative subtypes among young patients with breast cancer and analyzed the risk of late DR in patients without DR within 5 years after surgery. To our knowledge, this is the largest analysis for late DR of young patients with breast cancer with only ER-positive, *ERBB2*-negative subtype to date.

Several methods have been studied to estimate late DR in patients with hormone receptor–positive disease. Among these, Clinical Treatment Score 5 (CTS5) is a tool developed in the UK by Dowsett et al^20^ in 2018 and uses 4 variables to predict the risk of DR between 5 and 10 years.^20^ This DR prediction model was developed for postmenopausal patients in the ATAC and BIG1-98 cohorts, and the CTS5 calculator is available online for public use. However, various validation studies on CTS5 have shown that its predictive power decreases for premenopausal patients and that, under the same conditions, the risk of late DR tends to increase with age, which differs from our current understanding.^21,22,23,24^ Additionally, in a study by Lee et al^21^ analyzing data from 2605 Korean patients, including 1749 premenopausal patients, the predictive power of CTS5 for premenopausal patients decreased to an area under the curve of 61.8, whereas the area under the curve of CTS5 for postmenopausal patients was 72.7. This indicates that CTS5 may underestimate the risk and should be used with caution in premenopausal patients.^25^

There are several multigene assays widely used to estimate the risk of late DR or assess the benefits of chemotherapy in patients with hormone receptor–positive breast cancer.^26,27,28,29^ However, the accuracy of these genomic signatures can diminish based on myriad factors, such as patient age and node positivity.^30^ The TAILORx trial demonstrated that even within the intermediate risk category (recurrence score, 11-25), there was a range in which younger premenopausal patients derived greater benefit from chemotherapy compared with postmenopausal patients.^26^ These findings indicate that younger premenopausal patients may have a higher risk of DR even with the same clinicopathologic and genomic risk profiles, warranting careful consideration in adjuvant treatment.

Adding an OFS to the regimen has recently been identified as a critical factor in improving long-term survival in young premenopausal patients with hormone receptor–positive breast cancer receiving adjuvant ET. The 8-year follow-up of the SOFT/TEXT trials demonstrated significantly higher rates of both DFS and OS with the addition of OFS to tamoxifen rather than tamoxifen alone.^31^ Furthermore, 12-year follow-up results from the SOFT/TEXT trials demonstrated a significant reduction in recurrence risk with the use of aromatase inhibitors plus OFS in the younger premenopausal patients and patients with high-risk features, compared with tamoxifen plus OFS.^32^ In the subgroup analysis of this study, it was particularly noted that premenopausal patients younger than 35 years experienced a greater OS benefit from the use of aromatase inhibitors plus OFS, compared with tamoxifen plus OFS. When considering these findings with the results of our study, which suggest an increased risk of long-term DR in younger patients, it becomes evident that the use of OFS or aromatase inhibitors with OFS should be strongly considered, particularly in younger patients.

Additionally, recent advancements in adjuvant ET, particularly with the development of oral selective estrogen receptor degraders (SERDs), as investigated in the EMBER-4^33^ and CAMBRIA-1^34^ trials, offer promising alternatives for patients with ER-positive, *ERBB2*-negative breast cancer. These trials are exploring the efficacy of oral SERDs, such as imlunestrant and camizestrant, in reducing recurrence risk, potentially providing superior outcomes compared with standard ET. Through these research findings, we can anticipate not only the efficacy of oral SERDs as adjuvant therapy for patients with high-risk disease but also the potential benefits of prolonged use of OFS and extended therapy, especially for younger premenopausal patients.

### Limitations

Our study has several limitations. First, this study was a retrospective study of a single country and a single race. The study has the limitations of a retrospective analysis of data collected from multicenter sources. There were patients with follow-up loss and unknown data, especially on receptor expression and adjuvant treatment, because we collected data from patients who had surgery over a period from 2000 to 2011 with long-term follow up. Additionally, we were unable to collect *BRCA1/2* data for this cohort because genetic testing, including *BRCA1/2*, was not commonly performed in Korea at the time.^35^

The lack of data regarding the duration of OFS administration and the limited number of patients receiving OFS as an adjuvant therapy restricted the assessment of impact of OFS on oncologic outcomes. This is because at the time the patients in this study received adjuvant therapy, the results of the SOFT/TEXT trial had not yet demonstrated the usefulness of adding OFS for survival benefit.

In addition, we observed that more patients aged 21 to 35 years (15.9%) received less than 5 years of ET compared with those aged 36 to 40 years or aged 41 to 45 years (7.9% and 7.4%, respectively), and a lower proportion of patients aged 21 to 35 years received extended ET. While the retrospective data were not able to fully explain these differences, younger patients were more likely to face significant fertility concerns and attempts to conceive, which could have influenced their adherence to therapy.^36^ Additionally, the adverse effects of ET might have contributed to decreased adherence among younger patients.^37,38^

## Conclusions

In this retrospective cohort study, we found that age was an independent factor associated with late DR for young patients with breast cancer diagnosed with ER-positive, *ERBB2*-negative subtypes. These findings suggest that younger patients, particularly those 35 years or younger, exhibited worse oncologic outcomes compared with older groups, especially in terms of LRFS, DFS, and late DMFS. The results provided important insights into the management of young patients with breast cancer and the need for age-specific treatment approaches, potentially recommending incorporation of chemotherapy, extended ET, or addition of OFS in younger patients with high-risk disease to improve their oncologic outcomes.

## Notes

Supplement 1. **eTable 1.** Multivariable Analyses for Late Distant Recurrence with Continuous Value of Age **eTable 2.** Age Groups Categorized by 2-Year Intervals **eTable 3.** Cox Proportional Hazards Regression for Late Distant Recurrence according to 2-Year Intervals Age Group **eFigure 1.** Kaplan-Meier Survival Curves according to pN Stage **eFigure 2.** Kaplan-Meier Curves for Late Distant Recurrence-Free Survival According to Early Locoregional Recurrence **eFigure 3.** Kaplan-Meier Curves for Late Distant Recurrence-Free Survival According to Age Groups in Cohort Without Early Recurrence

Supplement 2.
Data Sharing Statement
