# Supplementary material for: Age and Late Recurrence in Young Patients With ER–Positive, ERBB2-Negative Breast Cancer
Source: JAMA Netw Open. 2024 Nov 7;7(11):e2442663. doi: 10.1001/jamanetworkopen.2024.42663 (PMC11544499; doi:10.1001/jamanetworkopen.2024.42663)
Supplement: Supplement 1. — eTable 1. Multivariable Analyses for Late Distant Recurrence with Continuous Value of Age eTable 2. Age Groups Categorized by 2-Year Intervals eTable 3. Cox Proportional Hazards Regression for Late Distant Recurrence according to 2-Year Intervals Age Group eFigure 1. Kaplan-Meier Survival Curves according to pN Stage eFigure 2. Kaplan-Meier Curves for Late Distant Recurrence-Free Survival According to Early Locoregional Recurrence eFigure 3. Kaplan-Meier Curves for Late Distant Recurrence-Free Survival According to Age Groups in Cohort Without Early Recurrence [file jamanetwopen-e2442663-s001.pdf]

# Supplemental Online Content

Shin DS, Lee J, Kang E, et al. Age and late recurrence in young patients with ER-positive, *ERBB2*-negative breast cancer. *JAMA Netw Open*. 2024;7(11):e2442663.
doi:10.1001/jamanetworkopen.2024.42663

**eTable 1.** Multivariable Analyses for Late Distant Recurrence with Continuous Value of Age

**eTable 2.** Age Groups Categorized by 2-Year Intervals

**eTable 3.** Cox Proportional Hazards Regression for Late Distant Recurrence according to 2-Year Intervals Age Group

**eFigure 1.** Kaplan-Meier Survival Curves according to pN Stage

**eFigure 2.** Kaplan-Meier Curves for Late Distant Recurrence-Free Survival According to Early Locoregional Recurrence

**eFigure 3.** Kaplan-Meier Curves for Late Distant Recurrence-Free Survival According to Age Groups in Cohort without Early Recurrence

This supplemental material has been provided by the authors to give readers additional information about their work.

© 2024 Shin DS et al. *JAMA Network Open.*

**eTable 1. Multivariable Analyses for Late Distant Recurrence with Continuous Value of Age**

| | Multivariable analysis | | |
|---|---|---|---|
| **Variables** | **Hazard Ratio** | **95% CI** | ***P* value** |
| **Age (continuous)** | | | |
| *per* 1-year increase | 0.91 | 0.88-0.93 | <.001 |
| **Pathologic T stage** | | | |
| T1 | 1 [Reference] | | |
| T2 | 1.68 | 1.18-2.40 | .004 |
| T3-T4 | 1.41 | 0.73-2.73 | .30 |
| **Pathologic N stage** | | | |
| N0 | 1 [Reference] | | |
| N1 | 1.51 | 0.92-2.47 | .10 |
| N2 | 2.02 | 1.15-3.56 | .01 |
| N3 | 1.63 | 0.76-3.48 | .21 |
| **Histologic grade** | | | |
| Grade 1 | 1 [Reference] | | |
| Grade 2 | 1.89 | 1.12-3.18 | .02 |
| Grade 3 | 1.85 | 1.05-3.27 | .04 |
| **Breast operation** | | | |
| BCS | 1 [Reference] | | |
| TM | 1.49 | 1.07-2.08 | .02 |
| **Axillary operation** | | | |
| SLNB | 1 [Reference] | | |
| ALND | 1.67 | 0.98-2.85 | .06 |

Abbreviations: CI, confidence interval; BCS, breast conserving surgery; TM, total mastectomy; SLNB, sentinel lymph node biopsy; ALND, axillary lymph node dissection.

© 2024 Shin DS et al. *JAMA Network Open.*

**eTable 2. Age Groups Categorized by 2-Year Intervals**

| Age group | No. (%) | Late distant recurrence | |
|---|---|---|---|
| | | No | Yes |
| Age ≤ 26 years | 20 (0.7) | 16 | 4 |
| 26 < Age ≤ 28 years | 20 (0.7) | 18 | 2 |
| 28 < Age ≤ 30 years | 49 (1.8) | 41 | 8 |
| 30 < Age ≤ 32 years | 81 (2.9) | 72 | 9 |
| 32 < Age ≤ 34 years | 118 (4.3) | 102 | 16 |
| 34 < Age ≤ 36 years | 215 (7.8) | 198 | 17 |
| 36 < Age ≤ 38 years | 307 (11.1) | 282 | 25 |
| 38 < Age ≤ 40 years | 445 (16.1) | 415 | 30 |
| 40 < Age ≤ 42 years | 503 (18.1) | 482 | 21 |
| 42 < Age ≤ 44 years | 663 (23.9) | 636 | 27 |
| Age = 45 years | 351 (12.7) | 341 | 10 |
| **Total, No. (%)** | 2772 (100) | 2603 (93.9) | 169 (6.1) |

© 2024 Shin DS et al. *JAMA Network Open.*

**eTable 3. Cox Proportional Hazards Regression for Late Distant Recurrence according to 2-Year Intervals Age Group**

| Comparison group | Hazard Ratio | 95% CI (Lower-Upper) | *P* value |
|---|---|---|---|
| Age ≤ 26 years | 7.40 | 2.32 - 23.60 | <.001 |
| 26 < Age ≤ 28 years | 3.12 | 0.68 - 14.23 | .14 |
| 28 < Age ≤ 30 years | 5.55 | 2.19 - 14.08 | <.001 |
| 30 < Age ≤ 32 years | 3.76 | 1.53 - 9.25 | .004 |
| 32 < Age ≤ 34 years | 4.80 | 2.18 - 10.58 | <.001 |
| 34 < Age ≤ 36 years | 2.80 | 1.28 - 6.12 | .01 |
| 36 < Age ≤ 38 years | 2.86 | 1.38 - 5.96 | .005 |
| 38 < Age ≤ 40 years | 2.40 | 1.17 - 4.90 | .02 |
| 40 < Age ≤ 42 years | 1.43 | 0.67 - 3.04 | .35 |
| 42 < Age ≤ 44 years | 1.42 | 0.69 - 2.93 | .35 |

Reference for Cox proportional hazards regression: Age = 45 years

Abbreviations: CI, confidence interval.

© 2024 Shin DS et al. *JAMA Network Open.*

## eFigure 1. Kaplan-Meier Survival Curves according to pN Stage

A. Locoregional recurrence-free survival

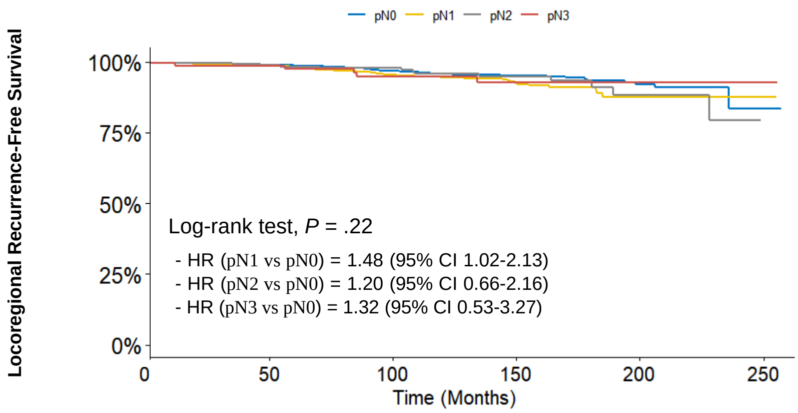


No. at risk

| | 0 | 50 | 100 | 150 | 200 | 250 |
|---|---|---|---|---|---|---|
| pN0 | 1713 | 1702 | 1342 | 458 | 109 | 2 |
| pN1 | 748 | 742 | 610 | 230 | 45 | 3 |
| pN2 | 223 | 221 | 194 | 87 | 27 | 0 |
| pN3 | 88 | 87 | 69 | 28 | 4 | 1 |

B. Disease-free survival

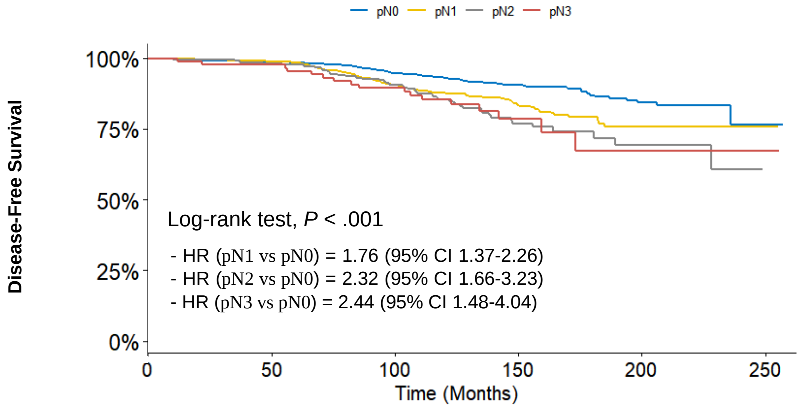


No. at risk

| | 0 | 50 | 100 | 150 | 200 | 250 |
|---|---|---|---|---|---|---|
| pN0 | 1713 | 1694 | 1313 | 432 | 100 | 2 |
| pN1 | 748 | 741 | 581 | 206 | 39 | 2 |
| pN2 | 223 | 219 | 183 | 74 | 23 | 0 |
| pN3 | 88 | 86 | 66 | 24 | 2 | 1 |

© 2024 Shin DS et al. *JAMA Network Open.*

C. Late distant metastasis-free survival

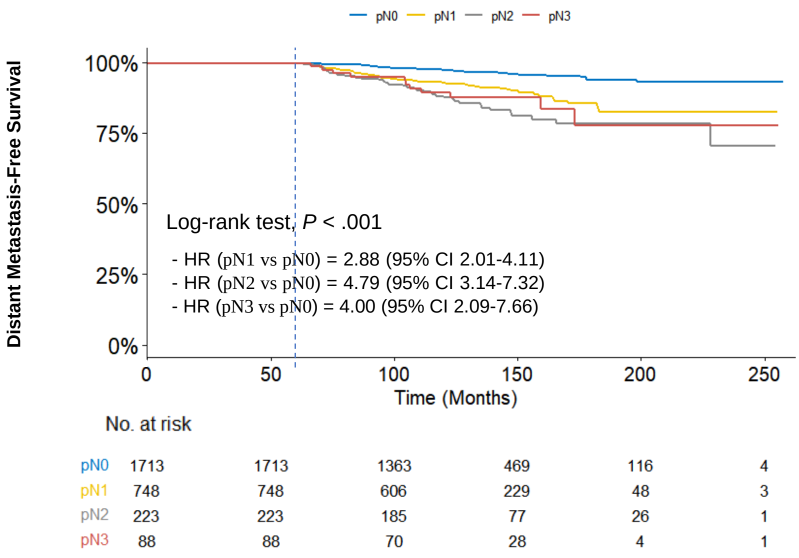


D. Overall survival

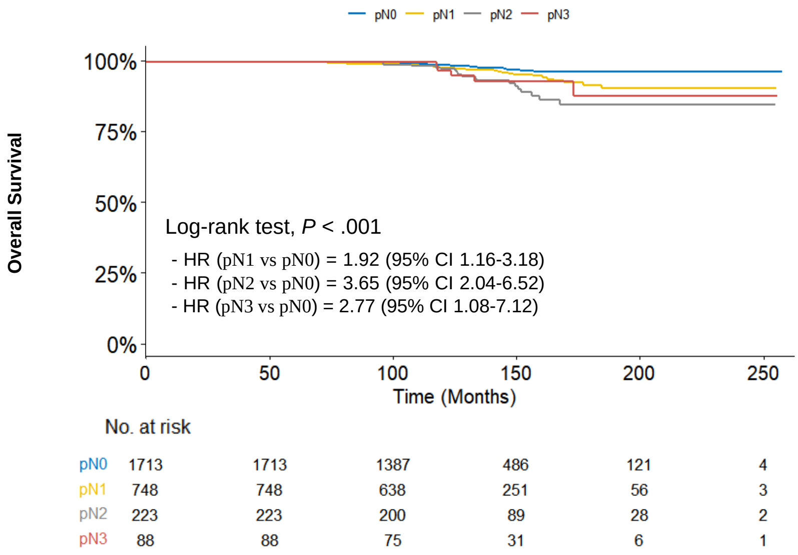


© 2024 Shin DS et al. *JAMA Network Open.*

**eFigure 2. Kaplan-Meier Curves for Late Distant Recurrence-Free Survival According to Early Locoregional Recurrence**

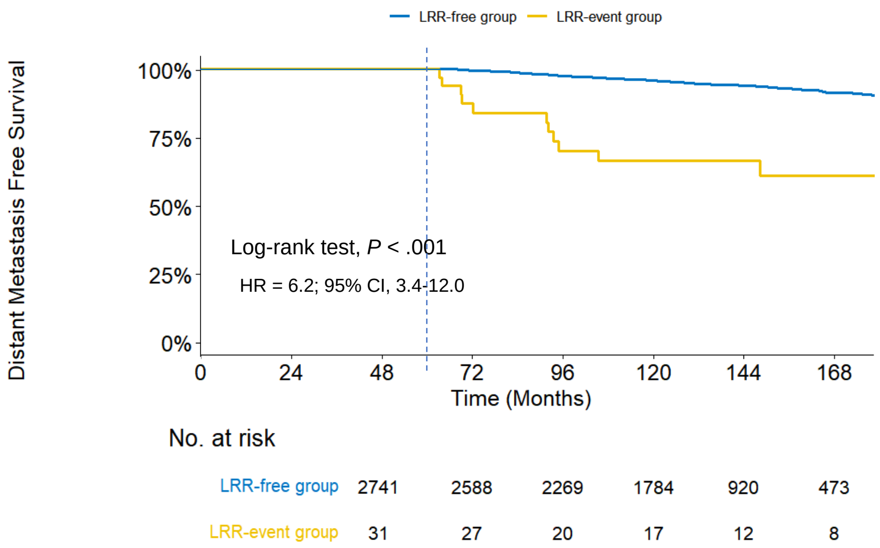


Abbreviations: LRR, locoregional recurrence; HR, hazard ratio.

Of the 2,772 patients, we analyzed late distant recurrence-free survival in 31 patients who experienced locoregional recurrence within the first 5 years versus 2741 patients who did not experience locoregional recurrence.

© 2024 Shin DS et al. *JAMA Network Open.*

**eFigure 3. Kaplan-Meier Curves for Late Distant Recurrence-Free Survival According to Age Groups in Cohort without Early Recurrence**

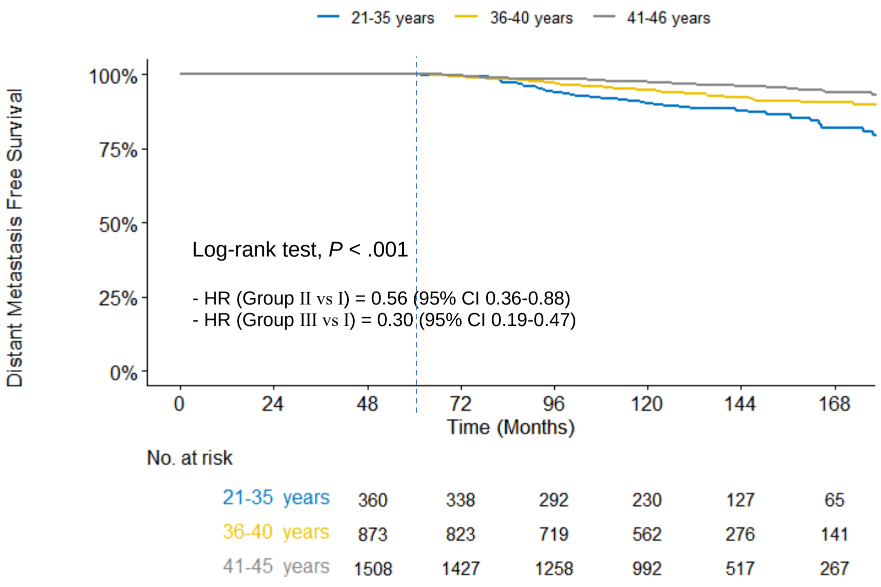


Abbreviations: HR, hazard ratio.

© 2024 Shin DS et al. *JAMA Network Open.*
