# Supplementary material for: Age and Late Recurrence in Young Patients With ER–Positive, ERBB2-Negative Breast Cancer
Source: JAMA Netw Open. 2024 Nov 7;7(11):e2442663. doi: 10.1001/jamanetworkopen.2024.42663 (PMC11544499; doi:10.1001/jamanetworkopen.2024.42663)
Supplement: Supplement 2. — Data Sharing Statement [file jamanetwopen-e2442663-s002.pdf]

Data Sharing Statement

Shin. Age and Late Recurrence in Young Patients With ER–Positive, ERBB2-Negative Breast Cancer. *JAMA Netw Open*. Published November 07, 2024.
doi:10.1001/jamanetworkopen.2024.42663

Data
Data available: Yes
Data types: Deidentified participant data
How to access data: Jai Min Ryu (jaimin.ryu@samsung.com) had full access to all the data in the study and takes responsibility for the integrity of the data and the accuracy of the data analysis.
When available: With publication

Supporting Documents
Document types: None

Additional Information
Who can access the data: researchers whose proposed use of the data has been approved
Types of analyses: for a specified purpose
Mechanisms of data availability: after approval of a proposal and with a signed data access agreement
Any additional restrictions: The data that support the findings of this study are available on request from the corresponding author, after proper revision of the data transfer agreement of the institutions and if ultimately allowed by Ethic Committees.
